# Antioxidant Potential and Oil Composition of *Callistemon viminalis* Leaves

**DOI:** 10.1155/2013/489071

**Published:** 2013-02-28

**Authors:** Muhammad Zubair, Sadia Hassan, Komal Rizwan, Nasir Rasool, Muhammad Riaz, M. Zia-Ul-Haq, Vincenzo De Feo

**Affiliations:** ^1^Department of Chemistry, Government College University, Faisalabad 38000, Pakistan; ^2^Department of Pharmacognosy, University of Karachi, Karachi 75270, Pakistan; ^3^Department of Pharmaceutical and Biomedical Sciences, University of Salerno, 84100 Salerno, Italy

## Abstract

The present study was designed to investigate the antioxidant potential and oil composition of *Callistemon viminalis* leaves. GC-MS analysis of the *n*-hexane extract revealed the presence of 40 compounds. Leaves contained appreciable levels of total phenolic contents (0.27–0.85 GAE mg/g) and total flavonoid contents (2.25–7.96 CE mg/g). DPPH radical scavenging IC_50_ and % inhibition of linoleic acid peroxidation were found to be in the ranges of 28.4–56.2 **μ**g/ml and 40.1–70.2%, respectively. The haemolytic effect of the plant leaves was found in the range of 1.79–4.95%. The antioxidant activity of extracts was also studied using sunflower oil as an oxidative substrate and found that it stabilized the oil. The correlation between the results of different antioxidant assays and oxidation parameters of oil indicated that leaves' methanolic extract, exhibiting higher TPC and TFC and scavenging power, was also more potent for enhancing the oxidative stability of sunflower oil.

## 1. Introduction

The genus *Callistemon* belongs to family Myrtaceae that has a great medicinal importance. The majority of *Callistemon* species are found in east and southeast of Australia. Phytochemical studies of different *callistemon* species revealed the presence of different monoterpenes, sesquiterpenes flavonoids. *Callistemon* species are used for forestry, essential oil production, farm tree/windbreak plantings, degraded land reclamation, and as bioindicators for environmental management and ornamental horticulture among other applications [1, 2]. Earlier phytochemical explorations of members of this genus resulted in the identification of C-methyl flavonoids, triterpenoids, and phloroglucinol derivatives [3–6]. Moreover, some medicinal properties like antimicrobial, antistaphylococcal, antithrombin and nematicidal activities and larvicidal and pupicidal values have been reported for this genus [7]. *Callistemon viminalis *(family: Myrtaceae) is a small tree or shrub with pendulous foliage, although some forms are more pendulous than others. This is an ornamental plant commonly known as bottlebrush that is found in several areas with the exception of localities extremely cold and dry. It is also found along the streets and in the botanical gardens [1–3]. *C. viminalis* is edible,and its leaves are a tea substitute and have a delightfully refreshing flavour and fragrance. Antihelminthic and antibacterial activities of *C. viminalis* various parts have been reported [8]. It has been used to prepare a hot drink locally referred to as “tea” for the treatment of gastroenteritis, diarrhea and skin infections [9]. As part of our studies on exploring medicinal flora of Pakistan for their compositional, nutritional and antioxidant potential [10–16], we studied the plant *C. viminalis* leaves to explore its antioxidant potential and oil composition.

## 2. Materials and Methods

### 2.1. Materials

The fresh leaves of the fully matured plant *C. viminalis* were collected on the basis of intensive review and ethnopharmacological information from Botanical Garden, University of Agriculture Faisalabad, Pakistan (A plane region latitude 31°-26′ N, longitude 73°-06′ E, and altitude 184.4 meters above main sea level) and further identified by a Taxonomist, Dr. Mansoor Hameed from Department of Botany, University of Agriculture Faisalabad, Pakistan. 

### 2.2. Sample Preparation

**Figure 1:**
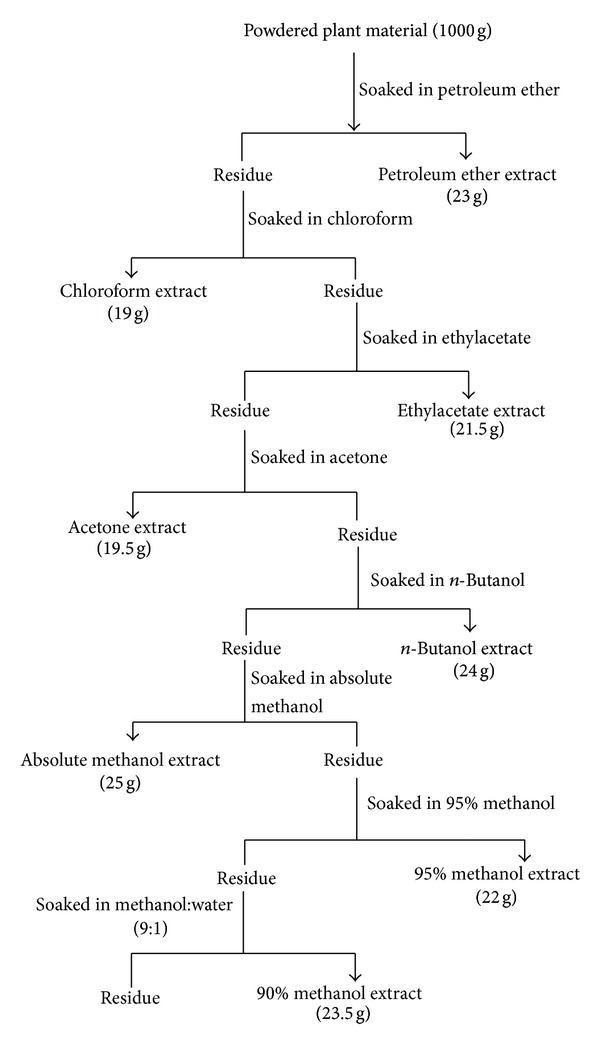
Schematic diagram showing preparation of extracts of *C. viminalis* leaves.

The plant leaves were washed with distilled water and then shade dried. The grinded fine powder of leaves was extracted with petroleum ether (2 × 2 L) for 6 h at room temperature. After filtering, the extract was concentrated through rotary vacuum evaporator (Eyela, Tokyo Rikakikai Co., Ltd., Japan). This process was repeated thrice to obtain a sufficient quantity of petroleum ether extract. The remaining plant residue was further extracted with other different polarity-based solvents and obtained successively chloroform, ethylacetate, acetone, *n*-butanol, absolute methanol, 95% methanol (95 : 5, methanol : water, v/v), and 90% methanol (90 : 10, methanol : water, v/v) extracts (Figure 1). All obtained extracts after drying were stored at −4°C till further analysis.

### 2.3. Preparation of *n*-Hexane Extract for GC-MS Analysis

The plant material (10 g of powdered leaves) was extracted with 300 mL *n*-hexane by using a Soxhlet apparatus for 6 h. The obtained *n*-hexane extract was filtered and evaporated by using a rotary evaporator and freeze dryer, respectively, to give the crude dried extract. The dried extract was stored at 4°C until used.

### 2.4. Evaluation of Antioxidant Activity

The plant leaves total phenolic contents (TPCs), total flavonoid contents (TFCs), DPPH radical scavenging IC_50_, and % inhibition of linoleic acid peroxidation were determined by the following methods described by Rasool et al [17]. Reducing the potential of the plant extracts was also determined [18]. 

### 2.5. Haemolytic Activity

Haemolytic activity of the plant was evaluated by following the already reported procedure [19].

### 2.6. Determination of Antioxidant Efficacy Using Sunflower Oil as Oxidation Substrate

#### 2.6.1. Stabilization of Sunflower Oil

The crude concentrated various extracts of the plant were separately added into the preheated (50°C), refined, bleached, and deodorized sunflower oil (SO) at concentration of 300 ppm (w/w). The oil samples were stirred for 30 minutes at 50°C for uniform dispersion. All oil samples were separately stabilized and stored in 100 mL airtight bottle. A control sample was also prepared (without extract) under the same set of analytical conditions. Samples were stored at room temperature. Synthetic antioxidant (BHT) was employed at its legal limit of 200 ppm to compare the efficacy of extracts. Stabilized and control oil samples (100 mL) were placed in dark brown airtight glass bottles with narrow necks and subjected to accelerated storage in an electric hot air oven (IM-30, Irmeco Gmbh & Co., Germany) at 60°C for 28 days. All oil samples were prepared in triplicate. Oil samples were taken after every 7-day intervals.

#### 2.6.2. Measurement of Oxidation Parameters of Sunflower Oil

The oxidative deterioration level was assessed by the measurement of peroxide value (PV), free fatty acids (FFAs) conjugate diene (CD), conjugate triene (CT), and *p*-anisidine values. Determination of the FFA and PV of stabilized and control sunflower oil samples were made following the AOCS Official methods Cd 8–53 and F 9a-44, respectively [20]. The oxidation products such as conjugated dienes and conjugated trienes were analyzed by following the IUPAC method II D.23 [21]. The absorbance was noted at 232 and 268 nm, respectively. The determination of the *p*-anisidine value was made following an IUPAC method II. D. 26 [21].

### 2.7. Oil Analysis

The GC-MS analysis of the *n*-hexane extract was performed using GC 6850 network gas chromatographic system equipped with 7683B series auto injector and 5973  i inert mass selective detector (Agilent Technologies USA). Compounds were separated on an HP-5 MS capillary column having 5% phenyl polysiloxane, a stationary phase with the column length 30.0 m, internal diameter 0.25 mm, and film thickness 0.25 *μ*m. The temperature of injector port was 300°C, and 1.0 *μ*L of sample was injected in the split mode with split ratio 30 : 1. The helium was used as the carrier gas at constant flow with the flow rate of 1.5 mL/min. The temperature program used for column oven was an initial temperature 150°C and held for 1 min, then ramped at a rate of 10°C/min up to 290°C, and finally held at this temperature for 5 min. The temperature of MSD transfer line was 300°C. For mass spectra determination, MSD was operated in electrom ionization (EI) mode, with the ionization energy of 70 eV, while the mass range scanned was 3–500 *m/z*. The temperature of ion source was 230°C and that of MS quadrupole 150°C. The identification of components was based on the comparison of their mass spectra with those of NIST mass spectral library [22, 23].

### 2.8. Statistical Analysis

Each sample was analyzed individually in triplicate, and data were reported as mean (*n* = 3 × 3 × 1)  ± standard deviation (*n* = 3 × 3 × 1). Data were analyzed by analysis of variance (ANOVA) using Minitab 2000 version 13.2 statistical software (Minitab Inc., Pennysylvania, USA).

## 3. Results and Discussion

### 3.1. Antioxidant Analysis

The yield (g/100 g) of various extracts from the plant *C. viminalis* leaves using different solvents ranged from 1.90% to 2.5%. The amounts of TPC and TFC (Table 1) from plant leaves in different solvent systems were found to be ranged from 0.27 to 0.85 GAE (mg/g of leaves extracts) and 2.25–7.96 CE (mg/g of leaves extracts), respectively. The ability of different solvents to extract TPC was found asfollows: absolute methanol > 90% methanol > chloroform > acetone > 95% methanol > ethylacetate > *n*-butanol > petroleum ether. The effect of different solvents on TFC values was found in the following order: absolute methanol > 95% methanol > chloroform > acetone > 90% methanol > ethylacetate > *n*-butanol > petroleum ether. These differences in the amount of TPC and TFC may be due to the varied efficiency of the extracting solvents to dissolve endogenous compounds. The leaves extracts exhibited different radical scavenging activity having IC_50_ value 28.4–56.2 *μ*g/mL. Absolute methanolic extract exhibited lowest IC_50_ (28.4 *μ*g/mL) followed by 95% methanol (34.1 *μ*g/mL), chloroform (38.2 *μ*g/mL), acetone (41.4 *μ*g/mL), 90% methanol (45.2 *μ*g/mL), ethylacetate (45.8 *μ*g/mL), *n*-butanol (52.1 *μ*g/mL), and petroleum ether (56.2 *μ*g/mL) extracts. The free radical scavenging activity of absolute methanol and 95% methanol extracts was superior to that of other solvent extracts. However, All extracts offered slightly less scavenging activity as compared to the synthetic antioxidant BHT (19.2 *μ*g/mL). The nature and amount of secondary metabolites of the plant cause the variation in free radical scavenging ability [24]. The free radical scavenging activity depends upon the chemical composition of extracts. Percent inhibition of linoleic acid oxidation ranged from 40.1% to 70.2%. The absolute methanolic extract exhibited the highest inhibition of linoleic acid oxidation (70.2%) and petroleum ether exhibited the lowest inhibition (40.1%). When the results of % inhibition of linoleic acid oxidation were compared with standard BHT (92.8%), all the samples showed significantly (*P* < 0.05) less antioxidant activity (Table 1). The order of inhibition of linoleic acid oxidation offered by various extracts of leaves was as follows: BHT > absolute methanol > 95% methanol > chloroform > acetone > 90% methanol > ethylacetate > *n*-butanol > petroleum ether. 

Results of the present study showed that among all the solvent extracts, absolute methanolic extract of plant leaves extracted the highest amount of TPC and TFC, which also demonstrated the highest antioxidant activity as measured by DPPH radical scavenging and inhibition of linoleic acid oxidation. This may be due to the high polarity of methanol, whereas, petroleum ether demonstrated the least antioxidant activity probably because of its low polarity. Previous reports [25, 26] also revealed that the methanolic extracts of plant materials offer more effective antioxidants. Antioxidant compounds were extracted from *Catharanthus roseus* shoots and found that methanol gave the maximum antioxidant yield [17]. Similar results were observed in the present investigations as methanol was most effective to extract antioxidative compounds.

### 3.2. Haemolytic Activity

Haemolytic activity was analyzed against human red blood cells (RBCs) using Triton X-100 as positive control. The % lysis of RBCs caused by the plant extracts was observed. Ethylacetate extract showed the highest haemolytic effect (4.95%) followed by petroleum ether (4.48%), 90% methanol (3.94%), chloroform (2.61%), 95% methanol (2.49%), acetone (2.33%), absolute methanol (2.03%), and *n*-butanol (1.79%) extracts, respectively. The haemolytic effect of *n*-butanol and absolute methanol extract was less then other extracts (Table 2). The mechanical stability of the erythrocytic membrane is a good indicator of the effect of various *in vitro* studies by various compounds for the screening of cytotoxicity. The percentage lysis of human erythrocytes was below 5.0% for all samples. All these results were in safe range. Though it can be expected that the plant extracts have a minor cytotoxicity [19, 27]. So pharmacologically this kplant may be safe to use for human beings as a source of potential drug.

### 3.3. Oil Analysis

The chemical compounds identified by GC-MS analysis of *n*-hexane extract presented in Table 3. The mass spectrum of each compound was compared with that in the NIST 05 library. Almost 40 different compounds were identified in *n*-hexane extract of plant leaves. The major compounds determined in the *n*-hexane extract were 2,5,5,6,8a-pentamethyl-trans-4a,5,6,7,8,8a-hexahydro-gamma-chromene (27.60%), (10E,12E)-10,12-tetradecadienyl acetate (11.62%), Z-7-tetradecenal (4.98%), 1,3-cyclohexadiene (3.97%), respectively. Some of the compounds were present in traces or less concentration as compared to other identified compounds. The *n*-hexane extract may have some fatty acids/methyl esters which may be implicated in some antioxidant and antimicrobial activities. During the literature review, it was found that the activities of some phytocomponents such as flavonoids, palmitic acid (hexadecanoic acid, ethyl ester and *n*-hexadecanoic acid), unsaturated fatty acid, docosatetraenoic acid, and octadecatrienoic acid as antimicrobial, antiinflammatory, and antioxidant activities [28]. Therefore, the chemical constituents found in may *C. viminalis *leaves play major roles in the biological activities and pharmacological properties.

### 3.4. Stabilization of Sunflower Oil

**Figure 2:**
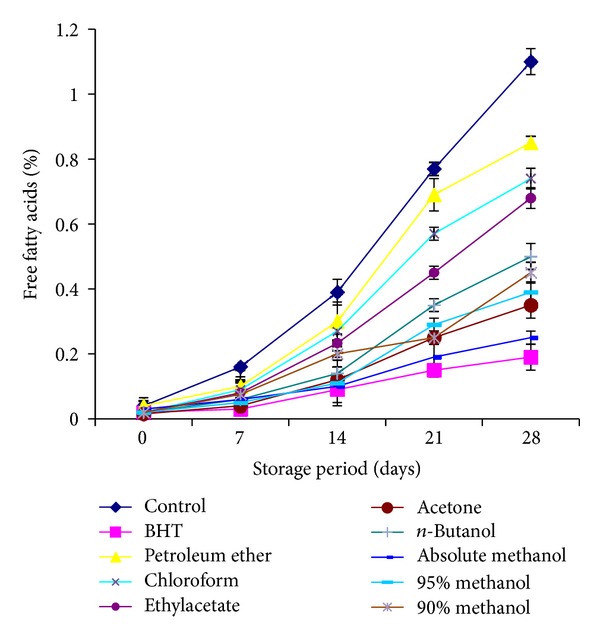
Free fatty acid contents (%) of sunflower oil stabilized with *C. viminalis* leaves extracts.

Formation of free fatty acids (FFAs) might be an important measure of rancidity of foods. FFAs are formed due to hydrolysis of triglycerides and may get promoted by the reaction of oil with moisture [29]. FFA content went on increasing with the increase in storage period for all the samples, but no regular pattern of increase could be observed. Control exhibited the highest FFA, while sunflower oil stabilized with BHT exhibited least (Figure 2). Initially, there was no increase in FFA of stabilized oil samples, but after seven days of storage, an increase that was observed showed the free fatty acid (FFA) contents of oil samples stabilized with leaves extract of *C. viminalis* under ambient storage conditions. All the oil samples stabilized with plant extracts were found to show a slow followed by a gradual increase in free fatty acid contents. The lower values of free fatty acid contents of stabilized oil samples than control indicated the effectiveness of leaves extracts as natural antioxidant in retarding the free fatty acid contents. Of all extracts, methanol extract was most efficient after BHT to inhibit the formation of FFA contents.

**Figure 3:**
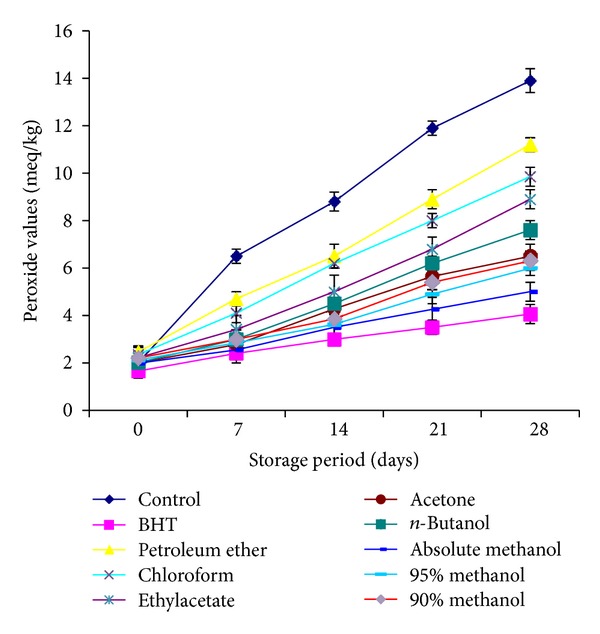
Peroxide values of sunflower oil stabilized with *C. viminalis* leaves extracts.

Peroxide value (PV) is usually used to evaluate the extent of primary oxidation products in oils. The highest PV was observed for control sample followed by petroleum ether > chloroform > ethylacetate > *n*-butanol > acetone > 90% methanol > 95% methanol > absolute methanol > BHT, respectively. All used extracts of the plant controlled peroxide value appreciably, revealing good antioxidant efficacy of extracts in stabilization of oil. A regular increase in PV as a function of storage time was observed for all the samples at all intervals. Initially, the difference in peroxide content of control and stabilized oil samples was not noticeable; it became significant (*P* < 0.05) just after heating up to one day (Figure 3).

**Figure 4:**
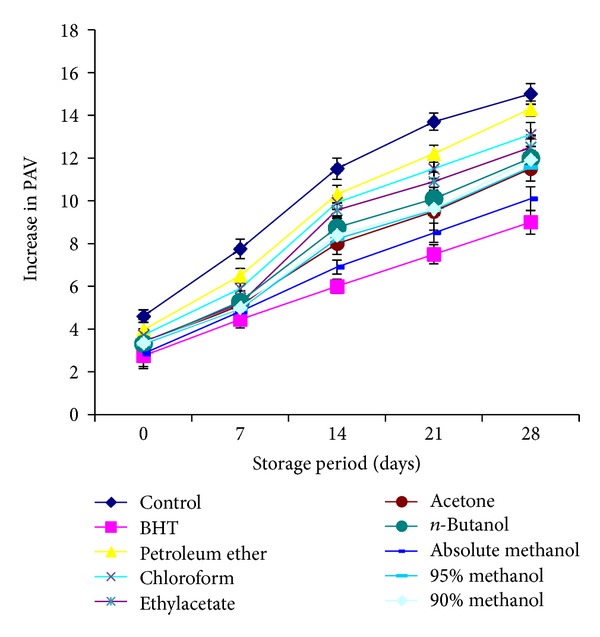
Relative increase in *p*-ansidine values of sunflower oil stabilized with *C. viminalis* leaves extracts.

The results for para-anisidine values (PAVs) which usually determine the amount of aldehyde in oils presented in Figure 4. The control sample showed the maximum increase in para-anisidine values indicating a higher rate of secondary product formation. A slow increase in PAV of stabilized sunflower oil as compared with the control indicating the antioxidant potential of the plant leaves. A decreasing order of stability of oil treated with different extracts of plant regarding para-anisidine values was found to be BHT > absolute methanol > acetone > 90% methanol > 95% methanol > *n*-butanol > ethylacetate > chloroform > petroleum ether > control.

**Figure 5:**
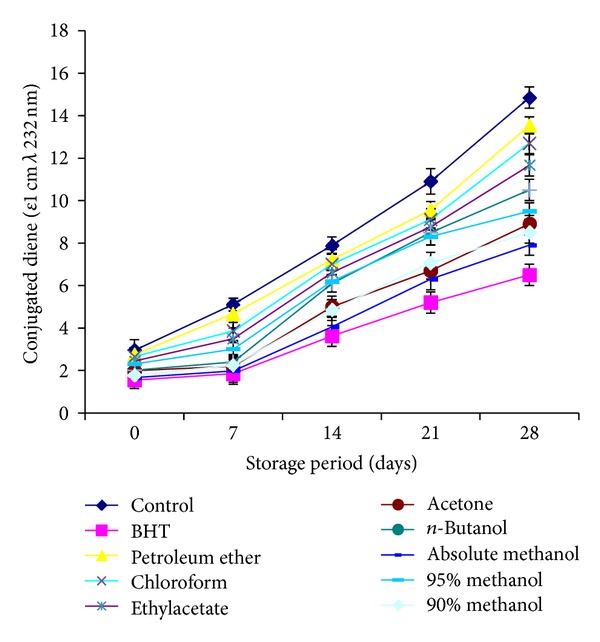
Relative increase in conjugated dienes content (CD) of sunflower oil stabilized with *C. viminalis* leaves extracts.

**Figure 6:**
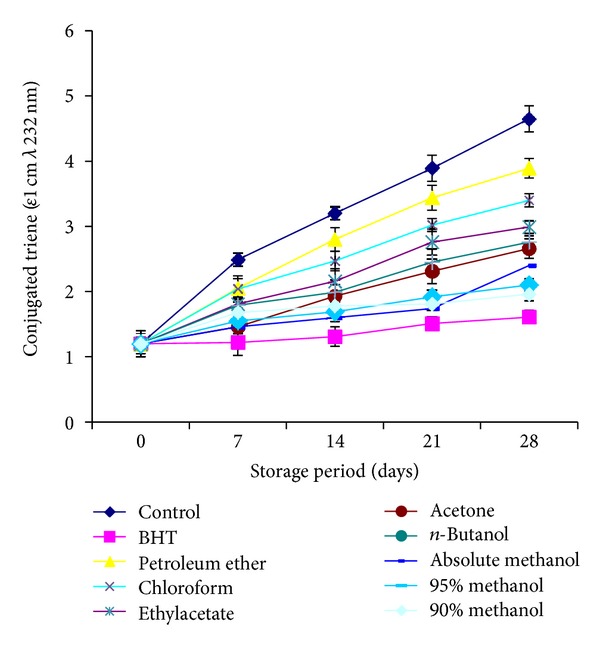
Relative increase in conjugated trienes content (CT) of sunflower oil stabilized with *C. viminalis* leaves extracts.

The formation of conjugated diene (Figure 5) and triene (Figure 6) analyzed for the control and stabilized sunflower oil, respectively. Highest contents were observed for control, indicating greater intensity of oxidation. 

The determination of CD and CT is a good measure of the oxidative state of oils [30] and thus a good indicator of effectiveness of antioxidants. CD and CT contents went on increasing with the increase in storage time. A slow increase in CD and CT of the stabilized sunflower oil as compared with those of the control indicated the antioxidant potential of the *C. viminalis* leaves. Stabilized sunflower oil conjugated diene and triene contens values were found to be in range of 1.66–13.54 and 1.2–3.89 (*ε*1 cm 232 nm), respectively. Absolute methanolic extract showed the lowest values and petroleum ether exhibited highest. All extracts played a prominent role for stabilization of sunflower oil but after standard BHT, methanol extract was most efficient to stabilize the oil.

## 4. Conclusions

The results of the present study concluded that the plant possessed considerable antioxidant potential, and it may also be used to stabilize the edible sunflower oil. Hence, the plant leaves investigated can be explored as a potential antioxidant source of natural origin. Cytotoxicity of plant extracts against human erythrocytes was checked, and it was in safe range, so the investigated plant may be safe use for pharmaceutical and natural therapies.

## Figures and Tables

**Table 1:** Antioxidant activity of  *C. viminalis* leaves^a^.

Extracts/fraction/standard	Yield (g/100 g)	Total phenolic contents^b^ (mg/g)	Total flavonoid contents^c^ (mg/g)	DPPH, IC_50_ (*μ*g/mL)	Inhibition in linoleic acid system (%)
Petroleum ether	2.30 ± 0.04	0.27 ± 0.001	2.25 ± 0.04	56.2 ± 0.54	40.1 ± 0.52
Chloroform	1.90 ± 0.02	0.71 ± 0.007	6.53 ± 0.06	38.2 ± 0.45	66.4 ± 0.77
Ethylacetate	2.15 ± 0.03	0.48 ± 0.005	5.11 ± 0.04	45.8 ± 0.51	54.1 ± 0.52
*n*-Butanol	2.40 ± 0.04	0.44 ± 0.004	3.96 ± 0.04	52.1 ± 0.62	48.2 ± 0.52
Acetone	1.95 ± 0.02	0.63 ± 0.007	6.43 ± 0.06	41.4 ± 0.52	56.8 ± 0.65
Absolute methanol	2.50 ± 0.04	0.85 ± 0.009	7.96 ± 0.08	28.4 ± 0.19	70.2 ± 0.77
95% methanol	2.20 ± 0.03	0.52 ± 0.006	6.69 ± 0.07	34.1 ± 0.41	68.2 ± 0.77
90% methanol	2.35 ± 0.03	0.75 ± 0.007	5.95 ± 0.07	45.2 ± 0.51	56.4 ± 0.61
BHT	—	—	—	19.2 ± 0.22	92.8 ± 0.91

^a^Values are mean ± S.D of three separate experiments.

^b^Total phenolic contents expressed as gallic acid equivalent.

^c^Total flavonoid contents are expressed as catechin equivalent.

**Table 2:** Percentage haemolysis caused by  *C. viminalis *leaves different extracts^a^.

Extracts	Percentage of haemolysis
Petroleum ether	4.48 ± 0.01
Chloroform	2.61 ± 0.02
Ethylacetate	4.95 ± 0.05
Acetone	2.33 ± 0.02
*n*-Butanol	1.79 ± 0.01
Absolute methanol	2.03 ± 0.02
95% methanol	2.49 ± 0.04
90% methanol	3.94 ± 0.03
Phosphate Buffer Saline (PBS)	0.00
Triton X-100	99.8 ± 1.01

^a^Values are mean ± S.D of three separate experiments.

**Table 3:** GC-MS analysis of  *n*-hexane extract of  *C. viminalis* leaves.

Peak number	Retention time	Compounds	% area
1	5.411	*n*-Octane	0.87
2	6.350	2-Methyloctane	0.62
3	7.754	o-Xylene	0.56
4	7.882	3-Methyloctane	0.68
5	8.964	*n*-Nonane	1.44
6	13.004	1,3-Cyclohexadiene	3.97
7	16.301	Undecane	1.07
8	19.590	*n*-Dodecane	1.02
9	22.354	2-Isopropyl-5-methylphenol	1.64
10	23.309	Exo-2-hydroxy cineole	1.26
11	24.266	Etyhl-5,9-dimethyl-2,4-decadienoate	3.95
12	25.413	3-Methyl-5-(2,6-dimethylheptyl)-1,5-pent-2-enolide	1.20
13	25.497	*n*-Tetradecane	1.27
14	26.880	4-Oxo-*β*-isodamascol	0.71
15	28.026	*n*-Pentadecane	0.62
16	28.170	2,4-Di-tert-butylphenol	2.75
17	28.391	Durohydroquinone	0.92
18	28.867	(10E,12E)-10,12-Tetradecadienyl acetate	11.62
19	29.027	2,5,5,6,8a-Pentamethyl-trans-4a,5,6,7,8,8a-hexahydro-gamma-chromene	27.60
20	29.783	(−)-Spathulenol	2.07
21	30.117	Hexadecane	2.34
22	31.919	10-Methylicosane	1.25
23	33.537	Heneicosane	3.09
24	33.583	Origanene	0.54
25	35.008	*n*-Nonadecane	0.62
26	35.837	Eicosanoic acid	3.37
27	36.382	*n*-Tetracosane	1.86
28	37.672	1,54-Dibromotetrapentacontane	0.49
29	37.791	Trans-phytol	1.05
30	38.074	Cis-9,cis-12-octadecadienoic acid	2.38
31	38.157	Z-7-Tetradecenal	4.98
32	38.411	Stearic acid	0.60
33	38.892	*n*-Tetratricoaconate	0.81
34	40.175	Ergost-7,22-dien-9,11-epoxy-3-ol, acetate(ester)	0.75
35	40.558	Furostan-12-one	2.37
36	42.576	Mono(2-ethylhexyl)phthalate	2.21
37	44.934	Trans-squalene	1.74
38	45.506	*n*-Hexatriacontane	0.50
39	47.309	*α*-Tocopherol-*β*-D-mannoside	0.82
40	49.184	Gamma-sitosterol	2.42
